# Supplementary material for: A Loop-Mediated Isothermal Amplification Assay for Rapid Detection of Pectobacterium aroidearum that Causes Soft Rot in Konjac
Source: Int J Mol Sci. 2019 Apr 19;20(8):1937. doi: 10.3390/ijms20081937 (PMC6514923; doi:10.3390/ijms20081937)
Supplement: Supplementary file 1 [file ijms-20-01937-s001.pdf]

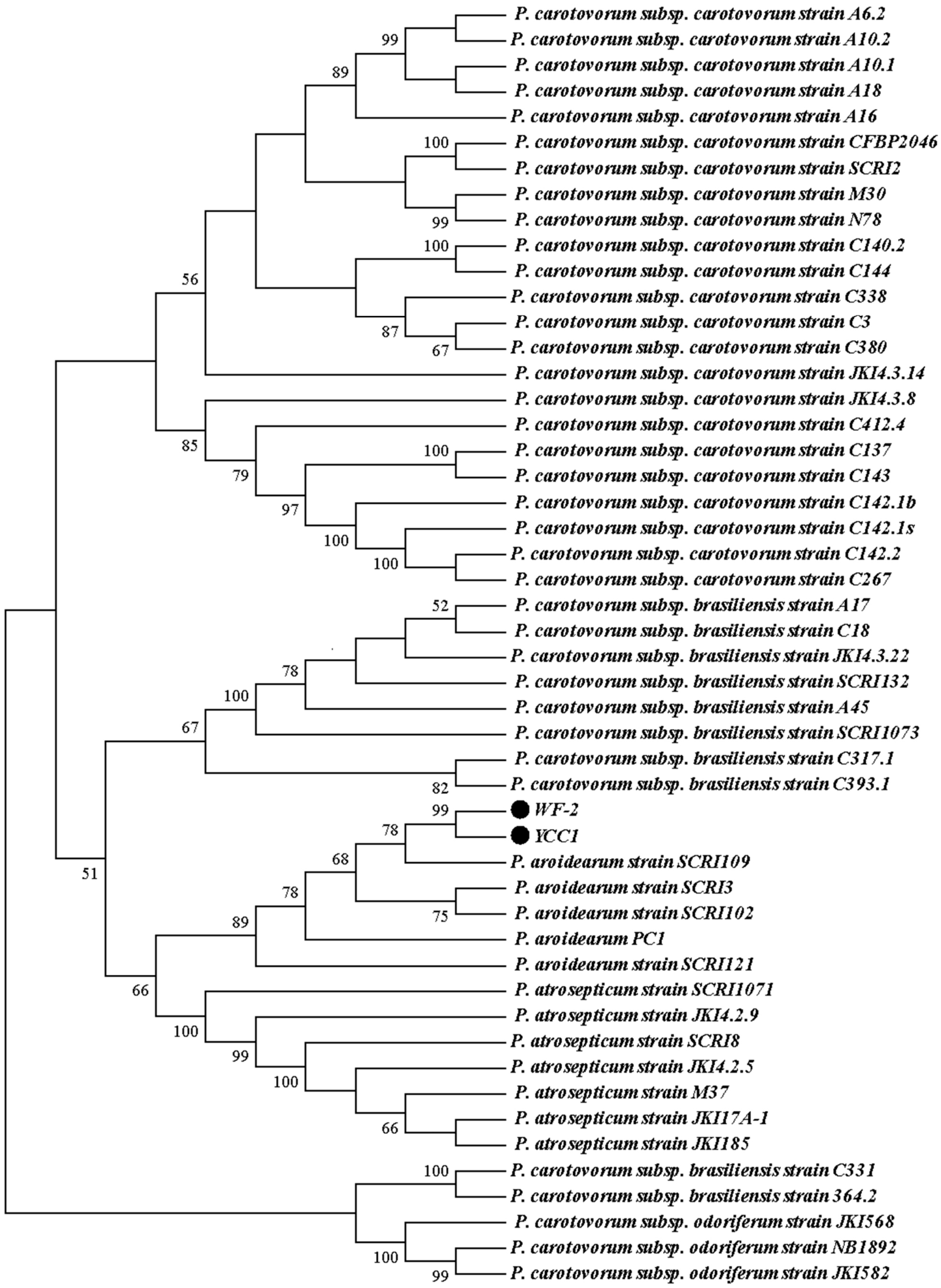

Figure S1. MLSA analysis based on eight housekeeping genes (*acnA*, *gabA*, *icdA*, *mdh*, *mtlD*, *pgi*, *proA* and *rpoS*) of the strains of four species of *Pectobacterium* plus our two isolate WF-2 and YCC1. The evolutionary history was inferred by using the maximum likelihood method based on the Tamura-Nei model. Bootstrap values from 1000 replicates are shown.

Table S1. Sources of the 50 species genomes required to build a local database.

Species	Strain	Data Size (Mb)	Genomic Origin[a]
Agrobacterium rhizogenes	NCPPB2659	2.76	NCBI
Agrobacterium tumefaciens	Ach5	2.66	NCBI
Bacillus amyloliquefaciens	CC178	3.73	NCBI
Bacillus megaterium	JX285	4.57	NCBI
Bacillus subtilis	QB928	3.95	NCBI
Burkholderia cepacia	LO6	5.76	NCBI
Clavibacter michiganense subsp. *sepedonicum*	Unknown	3.09	NCBI
Clavibacter michiganensis subsp. *michiganensis*	NCPPB382	3.14	NCBI
Dickeya chrysanthemi	M074	4.57	JGI
Dickeya dadantii	3937	4.19	NCBI
Dickeya dianthicola	RNS04.9	4.32	JGI
Dickeya paradisiaca	NCPPB2511	4.3	JGI
Dickeya solani	IPO2222	4.45	JGI
Dickeya zeae	EC1	4.15	JGI
Dyella japonica	A8	4.05	NCBI
Erwinia amylovora	CFBP1430	3.48	NCBI
Erwinia billingiae	Eb661	4.77	NCBI
Erwinia gerundensis	E_g_EM595	4.2	JGI
Erwinia iniecta	B149	4.61	JGI
Erwinia oleae	DAPP-PG531	4.5	JGI
Erwinia persicina	NBRC102418	4.64	JGI
Erwinia piriflorinigrans	CFBP5888	3.68	JGI
Erwinia pyrifoliae	DSM12163	3.7	NCBI
Erwinia tasmaniensis	ET199	3.57	NCBI
Erwinia tracheiphila	BuffGH	4.5	JGI
Erwinia typographi	M043b	5.41	JGI

Escherichia coli	CFT073	5.14	NCBI
Gemmatimonas aurantiaca	T-27	4.5	NCBI
Pantoea ananatis	LMG20103	4.41	NCBI
Pantoea stewartii subsp. *stewartii*	DC283	4.18	NCBI
Pectobacterium atrosepticum	JG10-08	4.61	NCBI
Pectobacterium betavasculorum	NCPPB2793	4.52	JGI
Pectobacterium carotovorum subsp. *actinidiae*	KKH3	4.66	JGI
Pectobacterium carotovorum subsp. *brasiliense*	BD225	4.59	JGI
Pectobacterium carotovorum subsp. *odoriferum*	NCPPB3841	4.43	JGI
Pectobacterium wasabiae	NCPPB3701	9.31	JGI
Planctomycetes bacterium	NH11	8.13	NCBI
Pseudomonas aeruginosa	PAO1	5.89	NCBI
Pseudomonas amygdali pv. *lachrymans*	NM002	5.65	NCBI
Pseudomonas putida	B6-2	5.88	NCBI
Pseudomonas syringae pv. *tomato*	DC3000	5.87	NCBI
Ralstonia solanacearum	EP1	3.61	NCBI
Rhizobium etli	N561	4.17	NCBI
Rhodanobacter denitrificans	2APBS1	4.01	NCBI
Serratia marcescens	UMH3	4.96	NCBI
Sphingomonas melonis	TY	3.85	NCBI
Xanthomonas campestris pv. *campestris*	B100	4.64	NCBI
Xanthomonas citri subsp. *citri*	TX160197	4.9	NCBI
Xanthomonas oryzae pv.*oryzae*	PXO145	4.55	NCBI
Xylella fastidiosa	3124	2.35	NCBI

[a] NCBI: https://www.ncbi.nlm.nih.gov/; JGI: https://genome.jgi.doe.gov/portal/.
